# Challenges and prospects of food science and technology education: Nepal's perspective

**DOI:** 10.1002/fsn3.173

**Published:** 2014-09-24

**Authors:** Ghanendra Gartaula, Bhaskar Mani Adhikari

**Affiliations:** 1 Department of Food Technology, GoldenGate International College, Tribhuvan UniversityKathmandu, Nepal; 2 National College of Food Science and Technology, Tribhuvan UniversityKathmandu, Nepal

**Keywords:** Challenges, education, food science and technology, Nepal, prospects

## Abstract

Food science and technology education has been running since four decades in Nepal. There is a very slow improvement in the profession. The job opportunities have always been threatened by insiders and outsiders. Academic institutions, government agencies, and food industries themselves are responsible for the quality of food science professionals. Novel and practical methods of teaching should be followed. The government and private organizations should facilitate the recruitment of food technologists. Constant prodding needs to be done for the establishment of a Council with more authority that could monitor all bodies associated with food science professionals.

## Introduction

The study which consists of the physical, biological, and chemical makeup of food is called food science. It covers the concepts underlying food processing and preservation. Various scientific disciplines inclusive of chemistry, engineering, microbiology, and nutrition are applied so that the safety, nutrition, wholesomeness, and availability of food are improved. The application of these scientific disciplines to the selection, preservation, processing, packaging, distribution, and consumption is the food technology. It includes analytical chemistry, biotechnology, engineering, nutrition, quality control, and food safety management too (IFT 2014).

Cooking is believed to be the original form of food processing, which was discovered about 2 million years ago by a distant ancestor (Wrangham 2009). Human beings thus first learned how to cook food, then how to transform, preserve, and store it safely. The experience-based technology of cooking food, transforming one food to another, its preservation, and storage led to modern food processing (Hall 1989; Floros 2008). Later, humans started the domestication of plants and land cultivation; and at the end of the last Ice Age, humans started eating meat by domesticating animals for food (Floros et al. 2010).

There has been a drastic change in the production-to-consumption food system. These days the food is largely safe, tasty, nutritious, abundant, diverse, convenient, and less costly and more readily accessible than ever before. Present-day food science and technology has contributed greatly to the success of this modern food system by including all of the sciences, viz. physics, chemistry, biology, materials science and engineering, biotechnology, microbiology, nutrition, toxicology, computer science, etc. to resolve nutritional deficiencies and enhance food safety. Nutrients can be preserved, essential vitamins and minerals can be supplemented, toxic principles can be removed, and foods can be designed to optimize health and reduce the risk of disease. Similarly, product loss and wastage can be reduced, and the global distribution can be expedited to allow seasonal availability of many foods (Floros et al. 2010).

At a time when the world was ready to celebrate IFT's 75th anniversary on 21–24 June 2014 at New Orleans, US, Nepalese food scientists and professionals gathered for the 7th National Conference on Food Science and Technology (Food Conference-2014, 13–14 June 2014). This clearly depicts the position of Nepal. However, this should not be taken as a discouragement as history tells it needed a lot of effort and dedication to bring the status to this stage. Different seminars and conferences were held in the past, namely Rice Milling Conference, 1969, National Seminar on Food Industries and Food Technology-I (NASOFIFT-I), 1984 (2041 BS), NASOFIFT-II, 1988 (2044 BS), National Food Convention, 1991, NASOFIFT-III, 1993 (2050 BS), and Food Conference, 2012. A resolution was passed in Food Conference, 2012 that Nepal Food Scientists and Technologists Association (NEFOSTA) should organize a national level conference in every 2 years. Hence, the continuation of this conference should be taken as a positive sign in the development of food science and technology.

## History of Food Science and Technology Education in Nepal

Food technology education was introduced in Nepal in 1973 (2030 BS). The program was started in Dharan with a certificate level course in food technology under the aegis of Institute of Applied Science and Technology of Tribhuvan University. The objective of this course was to produce middle-level manpower in food technology. This program was terminated after completing four academic years in 1979 (2036 BS), and in the same year the bachelor degree program B.Sc. Food Technology (three academic years) was started, and later changed to 4 years B. Tech. (Food) under the Institute of Science and Technology, Tribhuvan University (Katawal 2012; KC 2012). After 26 years of the establishment of the college in the public sector (Tribhuvan University), a college was opened in the private sector to run Tribhuvan University's B. Tech. (Food) program in 2006 (Subba et al. 2013). Now there are altogether 13 colleges in the country for the food technology program. Postgraduate and doctoral program in food technology was launched in 2001 and is being run by Central Department of Food Technology, Tribhuvan University, Dharan. There is one college in the private sector, Himalayan College of Agricultural Sciences and Technology (HICAST), affiliated to Purwanchal University which is running courses of M.Sc. Dairy Technology and M.Sc. Meat Technology since 2003. There are two colleges affiliated to the Council of Technical Education and Vocational Training (CTEVT) offering vocational courses (diploma level) in food technology. They produce middle-level technical manpower for food industries and organizations having quality control functions. Realizing the need of manpower in human nutrition, a bachelor degree program in nutrition and dietetics (B.Sc. Nutrition and Dietetics) was started in 2009 by the Central Campus of Technology, Dharan. The College of Applied Food and Dairy Technology (CAFODAT), Kathmandu has started an M.Sc. program in Nutrition and Dietetics in 2012 (KC 2012; Subba et al. 2013). The institutions that run courses on food science and technology are listed in Table1.

**Table 1:** Colleges offering food science courses in Nepal

S. no.	Name	Address
1	Central Campus of Technology, Tribhuvan University (TU)	Dharan
2	Lalitpur Valley College, TU	Lalitpur
3	National College of Food Science and Technology, TU	Kathmandu
4	Padmashree International College, TU	Kathmandu
5	GoldenGate International College, TU	Kathmandu
6	Sunsari Technical College, TU	Dharan
7	Dharan Multiple Campus, TU	Dharan
8	Pokhara Bigyan tatha Prabidhi Campus, TU	Pokhara
9	Birat Multiple College, TU	Biratnagar
10	Nilgiri College, TU	Itahari
11	Nagarik College, TU	Chitwan
12	Himalayan College of Agriculture Science and Technology, Purbanchal University (PU)	Kathmandu
13	College of Applied Food and Dairy Technology, PU	Kathmandu

As of 2012, the numbers of persons who received different degrees within the country are: PhD—2, M. Tech.—51, M.Sc.—18, B. Tech.—544, Diploma—48, and PCL—80. About 20 students have been already awarded PhD degree and 10 students are doing their PhD in different universities abroad. Ten PhD students are either employed or doing postdoctorate in different universities (Subba et al. 2013). Till date, almost all graduates are job holders in good positions.

## Opportunities

Food science and technology ensures the delivery of a safe, nutritious, and abundant food globally. It helps us to advance the food system, minimize risks, and maximize benefits. It can address specific issues throughout the food system. Many raw food materials and ingredients are transformed into consumable and safe foods that are available all round the year. Developments in food science and technology have enhanced food safety and have kept the quality consistent; provided reduction in nutrient deficiency-related diseases; reduced food waste; decreased home food-preparation time; lower household food costs; products specifically formulated to meet the nutritional needs of specific subpopulations; and efficient global food distribution (Floros et al. 2010).

Food science and technology is one of the very few courses in Nepal that promotes interactive learning methods. During the graduation phase, students get chances to broaden their knowledge and explore their possibilities. The course includes local field visits, industrial tour, and in-plant (industrial) training, aside from routine theory and practical classes. Local field visits increase interests and create more positive feelings toward that topic. Industrial tour across the country and industrial trainings provide opportunities to learn practically through interaction. During the in-plant training period, the students visit the industry and get insights regarding the working environment. The training which lasts more than a month improves their entrepreneurial ability, helps to come across real problems, and to find proper solutions. The training will provide industrial exposure to the students as well as help them to develop their career in high-tech industrial requirements. Toward the end of the study period, each student is required to do a dissertation independently. This will help to deepen and extend the knowledge of the chosen topic, helps to solve problems with confidence, and strengthens the student for large and complex research projects.

Consumers are increasingly demanding healthy foods. Hence, food science has better job prospects than any other industry. Worldwide, the areas of good job prospects are product development, quality control, and nutrition and food safety (Katsnelson 2012). In Nepal, most of the graduates are employed in food processing industries. They have joined services of universities, colleges, schools, food industry, government and semigovernment sectors, UN agencies, nongovernment organizations, projects, marketing companies, consultancy firms, etc. and their services include teaching, research, training, production, business management, quality control, nutrition-related studies, diet planning, quality audit, consultancy, industrial quality standards management, provide technical expertise, marketing, and so on. Some are working independently in their own industry and business and some do freelance jobs. Food technology graduates have largely fulfilled the needs of different food and nutrition-related sectors (KC 2012; Subba et al. 2013). A recent report on the status of food technology graduates (Guragain 2014) shows that the majority of the Nepalese graduates (33%) are absorbed by the food industries, followed by academic institutions (11%) and government sectors (10%). About 21% of the graduates are abroad, either pursuing their higher studies or working in food-related sectors. Very few (3%) are involved in development organizations, about 2% are in marketing sectors, and roughly 2% have established their own business. The remaining ones remain hidden in the profession due to either of the reasons as shift in profession, out of contact, joblessness, retirement, or death.

The right of every Nepalese to adequate food has been included in the Interim Constitution of Nepal, 2007 and clarified by the interim order of the Supreme Court of Nepal in September 2008. The Supreme Court published a key decision in April 2011 regarding the right to food in the country. The Interim Constitution recognizes food sovereignty and food security as a basic right (FAO 2014). As per P. Koirala, the spokesperson of Department of Food Technology and Quality Control (DFTQC) under Ministry of Agricultural Development, a proposal has been submitted to the Government of Nepal to recruit at least one food technologist in all of the 75 districts (P. Koirala, pers. comm., 4 July 2014). People have become more conscious about food safety due to the routine inspection of eateries, hotels, and industries, and awareness programs run by DFTQC, and other professional organizations like NEFOSTA and Food and Nutrition Awareness Centre Nepal (FONAC). This has demanded and created more opportunities to food science graduates.

The World Summit on Food Security (2009) recognized that by 2050, the global food production must increase by about 70% to feed the estimated 9 billion people (FAO 2009). This projection is expected to increase the annual consumption of nearly 1 billion metric tons of cereals for food and feed and 200 million metric tons of meat. This can be possible only by science-based improvements in the production of agricultural goods, application of food science and technology, and improvement in food supply systems (Floros et al. 2010). This, of course, certainly demands food scientists and technologists. Hence, the scope of food science graduates is increasing day by day.

## Challenges

With great opportunities, challenges come along. The developments in food science and technology have always been thwarted by both insiders and outsiders. To begin with, the need of amendments in syllabus has been the greatest challenge. There have been minor changes in the curriculum since the inception of the course. Since four decades the world food science has changed a lot and we are still in the same course designed in the 1970s. Important changes are necessary in the current curricula to meet new challenges for educating the future students in food science. Although the language of instruction and assessment for the course is English, the students find it difficult when it comes to speaking and writing. This is because English is not included as a subject in the curriculum. The syllabus is more theory oriented. The university has been very flexible in distributing license/affiliation to academic institutions without fulfilling criteria about physical infrastructures and sustainability. Many colleges are operating without proper and complete set of faculties, classroom, and laboratories. The teaching methodologies in many institutions are of the spoon-fed type. Students totally rely on lecture notes. Research culture is lagging in academic institutions. There are many traditional foods that await research and promotion but hindrances are the lack qualified manpower and well-structured laboratories. Lack of cooperation among academia and industries makes it difficult when it comes to conducting in-plant trainings and internships. This might be due to the flooding of interns from all academic backgrounds, be it science based or others. Postgraduation, lack of uniformity in pay scale motivates the mobility of fresh graduates from industries. This turnover has been a headache for industrialists. Time and again, the government claims that it has shortage of food technology manpower to fight the food safety and food security problems, but is reluctant to open vacancies for food research officers. Unlike other professions such as medicine, engineering, veterinary science, etc., food science and technology is devoid of Food Technology Council which could ease many of the aforementioned challenges.

## What Next

The government should develop and introduce a course about Food Science for Secondary and Postsecondary education. Important changes that are necessary in the current curricula of undergraduate and postgraduate courses should be amended to meet new challenges for educating the future students in food science. The curriculum should be at par with those of developed nations so that Nepalese graduates could be familiar with the global trends. Timely update of the curriculum should be done with rigorous discussions with industries, government authorities, and academics. More credit should be given to practical classes than the previous 75–25 approach. There should be regular monitoring of academic institutions to check if the operation of pilot plants, laboratories, and faculties are in place. Each institution should have a separate research unit for its faculties, and should encourage them to participate in research and publication process. Research can be done in a collaborative manner too. After completing preliminary studies here in Nepal, the sample may be microanalyzed in sophisticated laboratories of foreign universities. This will help to upgrade the research status of both the institutions.

Students should develop the habit of consulting different reliable sources to deepen their knowledge. Teaching faculties should encourage the use of student journals, motivate students for writing-to-learn assignments, practice cooperative and collaborative learning techniques, accommodation of varying learning styles, and methods to enhance problem-solving abilities and critical-thinking skills (Iwaoka et al. 1996). Interteaching may also be initiated. In a study, interteaching has been found as a novel approach to facilitate students of food science and nutrition. It is a 20- to 30-min student-to-student discussion which includes reciprocal peer tutoring, cooperative learning, and problem-based learning (Goto and Schneider 2010). Lectures should be accompanied by hands-on or visual explanations as described by Schmidt (2009). More emphasis should be given to practical demonstrations and that may be performed in food industries itself. But there must be mutual understanding among them.

Fresh graduates should keep in mind that experience, rather than money, counts. They should develop other skills such as communication, teamwork, honesty, sincerity, and flexibility, to name a few. NEFOSTA should work together with the likes of the Institute of Food Technologists to build bridges between food scientists around the world. Food science and technology professionals must work together with others—the food industry, research institutions, and those in the regulatory and public policy communities. A Council must be established that could monitor academic institutions, persuade government agencies, and poke industries for the betterment of food science professionals. There must be huge investment in basic and applied research, education, and extension.

## Conflict of Interest

None declared.
